# The Tripod for Bacterial Natural Product Discovery: Genome Mining, Silent Pathway Induction, and Mass Spectrometry-Based Molecular Networking

**DOI:** 10.1128/mSystems.00160-17

**Published:** 2018-03-27

**Authors:** Daniela B. B. Trivella, Rafael de Felicio

**Affiliations:** a Brazilian Biosciences National Laboratory (LNBio), Brazilian Center for Research in Energy and Materials (CNPEM), Campinas, Sao Paulo, Brazil

**Keywords:** bacterial secondary metabolites, biosynthesis, cryptic gene clusters, drug discovery, genome mining, mass spectrometry, microbial secondary metabolites, molecular networking, natural products

## Abstract

Natural products are the richest source of chemical compounds for drug discovery. Particularly, bacterial secondary metabolites are in the spotlight due to advances in genome sequencing and mining, as well as for the potential of biosynthetic pathway manipulation to awake silent (cryptic) gene clusters under laboratory cultivation. Further progress in compound detection, such as the development of the tandem mass spectrometry (MS/MS) molecular networking approach, has contributed to the discovery of novel bacterial natural products. The latter can be applied directly to bacterial crude extracts for identifying and dereplicating known compounds, therefore assisting the prioritization of extracts containing novel natural products, for example. In our opinion, these three approaches—genome mining, silent pathway induction, and MS-based molecular networking—compose the tripod for modern bacterial natural product discovery and will be discussed in this perspective.

## Abstract (precis)

Natural products are the richest source of chemical compounds for drug discovery. Particularly, bacterial secondary metabolites are in the spotlight due to advances in genome sequencing and mining, as well as for the potential of biosynthetic pathway manipulation to awake silent (cryptic) gene clusters under laboratory cultivation.

## PERSPECTIVE

**FIG 1:**
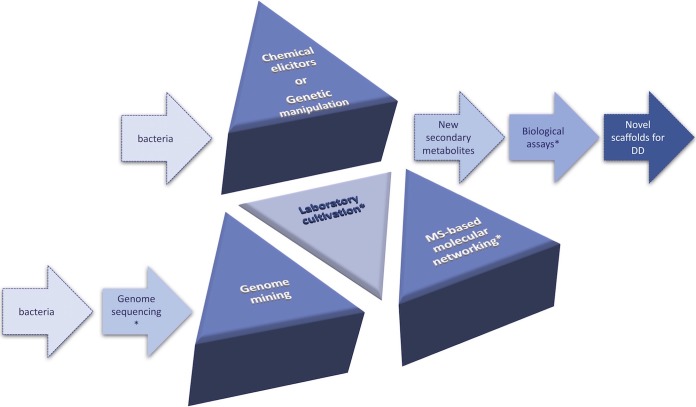
The tripod for modern natural product-based drug discovery. Genome mining, MS-based molecular networking, and growth conditions to elicit secondary metabolism as central strategies to drive new natural scaffold discovery for drug development. Asterisks indicate steps that can be done in miniaturized (e.g., 384-well plates) and automated scales.

In recent years, there has been a joint effort of the scientific community to address the bottlenecks in the discovery of new bioactive natural products (NP). Genome sequencing and mining, genetic or chemical manipulation of microbial growth, and mass spectrometry (MS)-based metabolomics are, in our opinion, the main evolving areas in this field and compose the tripod for modern natural product discovery (Fig. 1).

Natural products (NP) are the richest source of chemical compounds for drug discovery, comprising 65% of all small-molecule approved drugs (1). Particularly, compounds of microbial origin play a central role in this context, especially in the therapeutic areas of cancer and infectious diseases and as immunomodulatory drugs (1, 2). Among microbes, bacterial secondary metabolites are in the spotlight due to their simple genome organization and to the advances in genome sequencing and mining (3), as well as for their—in theory—facile biosynthetic pathway manipulation and laboratory cultivation (4).

However, natural product research is not an easy science, and obtaining secondary metabolites, even from bacteria, can be challenging and cost limiting for industry (2). Before the genome sequencing and mining revolution, less than 10% of the genetic capacity of well-known antibiotic-producing bacteria (such as those from the genus *Streptomyces*) was known, as were their secondary metabolites. If not stimulated, the biosynthetic potential of bacteria remains hidden under artificial cultivation (5, 6) and therefore unexplored. Furthermore, the yield of a target metabolite can be very low, unless stimulated by artificial methods. Another drawback of natural product discovery is that, until recently, time- and sample-consuming steps were necessary for compound isolation and identification, resulting in high rates of natural product rediscovery and massive strain cultivation and extraction (7).

Recent advances in each leg of the tripod for modern natural product discovery have increased the rate of new discoveries. A brief introduction and examples will be given below as a historical perspective.

### Genome mining.

**FIG 2:**
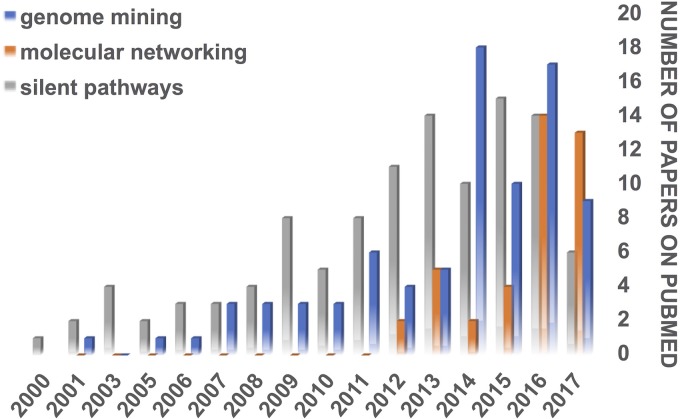
Use of genome mining, MS-based molecular networking, and investigation of silent (cryptic) biosynthetic pathways in scientific reports available on PubMed in the last 17 years. In this search, the terms “genome mining” and “bacteria” and “secondary metabolite or natural product” were used for the genome mining query, the term “MS molecular networking” was used for the MS molecular networking query, and the terms “bacteria” and “cryptic or silent” and “bacterial natural products” or “bacterial secondary metabolites” were used for the silent pathways query. Review articles were excluded in all queries. “Vaccine” was further excluded in the genome mining and silent pathways queries, “fungi” was excluded for the genome mining query (to focus on bacterial metabolites), and “proteomics” was excluded in the MS molecular networking search. Articles were individually reviewed for entering in the statistics.

In the last few decades, genome sequencing technologies have evolved, making it cheaper and faster to obtain a whole bacterial genome, for example. This technological revolution was followed by the development of computational tools to assemble and analyze genome sequences. Please see reference 3 for a complete review on this theme. Genome mining, the process of extracting information from genome sequences, has become a central approach in microbial natural product discovery, especially if the producing organism is a bacterium (Fig. 2).

Biosynthetic gene clusters (BGCs) are the core organization of bacterial biosynthetic pathways at the genome level. BGCs generally code for multidomain enzymes, such as polyketide synthases (PKS) and nonribosomal peptide synthases (NRPS), transporters, and decorating enzymes (such as halogenases, oxidases, and cyclases). BGC expression is regulated at the transcriptional level, and regulatory mechanisms are frequently found flanking the BGC (8, 9).

However, predictive bioinformatics still lacks enough information about key natural biosynthetic strategies to be able to mine an entire bacterial genome and to predict its biosynthetic products. The very discovery of the bioactive natural product, originating from either a particular bacterium or a biosynthetic pathway, remains a challenging process; nonetheless, much has evolved over the last decade. For example, evolving techniques for genetic manipulation and heterologous expression of full gene clusters of *Proteobacteria* in model organisms (e.g., *Saccharomyces cerevisiae* or *Bacillus subtilis*) have enabled the understanding of the biosynthesis of secondary metabolites in the phylum *Proteobacteria*. This is prompting the discovery of new biosynthetic and regulatory strategies in these microorganisms (8). Still, many biosynthetic enzymes are unprecedented for their catalytic activities and display only distant homologues or orthologues with known three-dimensional structures and validated catalytic mechanisms (as an example, please see reference 10). Even in well-known secondary metabolite producers, the organization of BGCs coding for secondary metabolites is being reviewed, and unexpected biosynthetic setups are being found (11). This new information is constantly incorporated into the bioinformatics platforms for mining bacterial genomes and extracting the candidate secondary metabolites that these organisms can produce and vice versa (3). Therefore, we expect that genome mining will increasingly contribute to secondary metabolite discovery.

### Awakening silent gene clusters.

Currently, many compounds are still discovered using traditional natural product chemistry, which includes chemical library assembly, screening, active compound isolation, and structure determination. As mentioned above, secondary metabolites can be produced by the bacteria only after stimulation. Therefore, mimicking the microbial native environment with chemical or physical factors is a strategy to stimulate the production of secondary metabolites. This area of research gained more attention after the year 2000, as illustrated by the increasing number of scientific papers focusing on silent (cryptic) biosynthetic pathways (Fig. 2). Biotic (such as cocultivation of the target organisms with potential competitor organisms) and abiotic (physical and chemical elicitors of the secondary metabolism) factors have been used with success to enhance the diversity and quantity of secondary metabolites produced by a target microorganism (5, 6, 12).

From 2000, the process of activating silent gene clusters was aided by genome mining and new methods in molecular biology. For example, plug-and-play vectors were constructed and used to clone entire gene clusters, removing negative regulators of the target gene cluster expression (13). Further, genes conferring self-resistance to a given metabolite were mined and used to detect BGCs and their encoded natural product’s biological target (reviewed in references 3 and 8. Reporter gene constructs using *lacZ* or enhanced green fluorescent protein (eGFP) have also been used to screen chemical elicitors of a target BGC (12). Altogether, these studies prompted the discovery of small molecules or molecular strategies capable of awakening silent BGCs in bacteria and fungi under laboratory cultivation.

### MS/MS molecular networking.

MS-based metabolomics is the technique of choice for rapid detection and dereplication of secondary metabolites in microbial cultures. Indeed, the latter cultures can be directly analyzed without the need for purification/isolation, and large data sets can be processed at once. Tandem MS (MS/MS) molecular networking has evolved over the last 5 years (Fig. 2) to successfully assist mining of natural product chemical collections; compound identification; and prioritization of microbial strains for metabolite isolation, biological investigation, and genome sequencing and mining (14). Briefly, MS-based molecular networking is an approach that organizes MS/MS data based on spectral similarity. Since a given spectrum is characteristic of a given chemical structure, it is possible to organize known and unknown compounds based on their structural similarity. Therefore, MS-based molecular networking is a powerful tool for the dereplication of complex chemical samples, such as those of natural products (7). Moreover, this technique is compatible with the Global Natural Products Social Molecular Networking (GNPS), an open-access database for organization and sharing of raw, processed, or identified MS/MS spectral data (14). In this way, combining molecular networking and GNPS can bolster the identification of specific chemical classes and compounds and assist the prioritization of samples for further investigation (15).

### Present state and perspectives of the tripod for bacterial natural product discovery.

As mentioned above, natural products are the richest source of new chemical scaffolds for the development of drugs for clinical use. We expect that combining the previously discussed technologies with high-throughput genome sequencing efforts and biological screening will further promote original findings in chemical scaffold discovery for drug development. In this context, our group is progressing toward this goal by using the pipeline illustrated in Fig. 1. In doing so, we expect to find new pharmaceutically relevant compounds capable of being produced by bacteria and yet hidden in their genome due to the presence of silent BGCs. In summary, we believe that the integration of genomic exploration with microbial metabolite production and detection will impact the field of natural product drug discovery and pharmaceutical development over the next few years.
